# A Mega-Analysis of Personality Prediction: Robustness and Boundary Conditions

**DOI:** 10.1093/geroni/igab046.2157

**Published:** 2021-12-17

**Authors:** Emorie Beck, Joshua Jackson

**Affiliations:** 1 Northwestern University Feinberg School of Medicine, Chicago, Illinois, United States; 2 Washington University in St. Louis, St. Louis, Missouri, United States

## Abstract

Decades of studies identify prospective associations between personality characteristics and life outcomes. However, previous investigations of personality characteristic-outcome associations have not taken a principled approach to sampling strategies to ensure the robustness of personality-outcome associations. In a preregistered study, we test whether and for whom personality-outcome associations are robust against selection bias using prospective associations between 14 personality characteristics and 14 health, social, education/work, and societal outcomes across eight different person- and study-level moderators using individual participant data from 171,395 individuals across 10 longitudinal panel studies in a mega-analytic framework with propensity score matching. Two findings emerged: First, personality characteristics remain robustly associated with later life outcomes. Second, the effects generalize, as there are few moderators of personality-outcome associations. In sum, personality characteristics are robustly associated with later life outcomes with few moderated associations. We discuss how these findings can inform studies of personality-outcome associations.

